# Supplementary material for: Investigating the influence of the physical environment on psychiatric nurses wellbeing and professional interactions: A convergent parallel mixed-method study protocol
Source: PLoS One. 2026 Jan 15;21(1):e0340429. doi: 10.1371/journal.pone.0340429 (PMC12806848; doi:10.1371/journal.pone.0340429)
Supplement: S1 File — (DOCX) [file pone.0340429.s001.docx]

**S1 File. Reporting Guidelines Checklists (GRAMMS, STROBE, COREQ)**

- [Good Reporting of A Mixed Methods Study (GRAMMS) checklist](#gkx5u9rvtoyg)
- [STROBE Statement—checklist of items that should be included in reports of observational studies](#vyn14v57jrm)

- [COREQ (COnsolidated criteria for REporting Qualitative research) Checklist](#kix.n70d45ykkhu6)

Good Reporting of A Mixed Methods Study (GRAMMS) checklist

| **No.** | **Guideline** | **Manuscript Reference** |
| --- | --- | --- |
| 1 | Describe the justification for using a mixed methods approach to the research question | Existing literature reveals gaps indicating that, while there is evidence of the environment's impact on human performance, there is a lack of sufficient research specifically targeting psychiatric nurses in psychiatric hospitals. - See **Introduction** section, **Effects of the Physical Environment on Healthcare Workers** subsection  Rationale for employing a mixed-method approach - gaining in-depth understanding of the environmental effect, because human factor is involved, and objective measures are not sufficient for understanding perception and well-being - See **Introduction** section, **Significance of Mixed Methods Approach** subsection |
| 2 | Describe the design in terms of the purpose, priority and sequence of methods | *Purpose of the design* - See **Methods and Analysis** section, **Study Design** subsection  *Priority* - See **Methods and Analysis** section, **Study Design** subsection  *Sequence of methods* **-** Parallel design - See **Methods and Analysis** section, **Timeline** subsection |
| 3 | Describe each method in terms of sampling, data collection and analysis | *Quantitative methods:*  *Sampling* - See **Methods and Analysis** section, subsections: **Study Setting***,* **Study Participants** and **Timeline**  *Data Collection* - See **Methods and Analysis** section, subsections: **Study Design***,* **Timeline** and **Quantitative Data**  Analysis - See **Methods and Analysis** section, **Data Analysis** subsection  *Qualitative methods:*  *Sampling* - See **Methods and Analysis** section, subsections: **Study Setting***,* **Study Participants** and **Timeline**  *Data Collection* - See **Methods and Analysis** section, subsections: **Study Design***,* **Timeline** and **Qualitative Data**  *Analysis* - See **Methods and Analysis** section, *Data Analysis* subsection |
| 4 | Describe where integration has occurred, how it has occurred and who has participated in it | *Where* - See **Methods and Analysis** section, subsections: **Study Design** and **Data Analysis**  *How* - See **Methods and Analysis** section, subsections: **Study Design** and **Data Analysis**  *Who* - The integration procedures will be conducted by two researchers involved in the study and reviewed by three research team members. |
| 5 | Describe any limitation of one method associated with the present of the other method | Integrating qualitative and quantitative data may lead to conflicting findings. For example, HRV may indicate low stress, while qualitative data reveal high levels of reported anxiety among participants. Resolving these discrepancies requires careful interpretation and contextual understanding.  HRV and light dosage measurements may be taken at specific times or over certain periods, while qualitative data from interviews or observations can capture broader experiences and contextual factors that are not easily correlated with the quantitative measures. Therefore, thematic analysis is used to extract qualitative themes that can be directly linked to specific HRV or light exposure measurements.  Described limitations are addressed with the mixed-method approach and comprehensive data collection and analysis, in order to minimize conflicting findings and avoid challenges with the interpretation of results. |
| 6 | Describe any insights gained from mixing or integrating methods | Qualitative data from observations and interviews capture participants' experiences, while quantitative measures like HRV and light dosage provide objective insights into physiological responses and environmental conditions. This combination helps identify correlations between stress levels and environmental factors, revealing patterns that may not emerge from either method alone and leading to more informed recommendations for improving psychiatric nurses' well-being and their work environment.  See **Methods and Analysis** section, **Study Design** subsection |

NA: Not applicable; Note: Since we are reporting on an ongoing mixed methods study, some criteria, recommendations, or items may not be complete, applicable or fulfilled (e.g., results and discussion sections).

STROBE Statement—checklist of items that should be included in reports of observational studies

|  | **Item No.** | **Recommendation** | **Relevant text from manuscript** |
| --- | --- | --- | --- |
| **Title and abstract** | 1 | (*a*) Indicate the study’s design with a commonly used term in the title or the abstract | Done. Please see the title page. Also please see the **Abstract**, section **Methods and Analysis**. We indicated the application of convergent parallel study design. |
|  |  | (*b*) Provide in the abstract an informative and balanced summary of what was done and what was found | Done. Please see **Abstract**. In the **Methods and Analysis** section we explain what is planned to be conducted in convergent parallel design study. We explain that the study focuses on understanding how environmental features impact psychiatric nurses' well-being and stress levels by integrating qualitative and quantitative data, including heart-rate variability, light exposure, sleep patterns, movements, and interactions, collected through wearable sensors, surveys, and observations. |
| **Introduction** | | |  |
| Background/rationale | 2 | Explain the scientific background and rationale for the investigation being reported | Done. Please see the **Introduction** section. The subsections cover the following topics: 1) Workplace Stress in Psychiatric Nursing, 2) Nurse-Patient Interactions in Psychiatric Hospitals, 3) Effects of the Physical Environment on Healthcare Workers and 4) Significance of Mixed Methods Approach. |
| Objectives | 3 | State specific objectives, including any prespecified hypotheses | Done. Please see section **Introduction**, subsection **Research Questions and Expected Outcomes**. We indicated a research aim which is to explore the connection between the physical work environment and the stress levels of nursing staff in psychiatric hospitals.. We also indicated research questions, which are the following:   1. Is there an association between the characteristics of the physical environment and stress levels of the nursing staff in psychiatric hospitals? 2. How do nurses working in psychiatric hospitals perceive their working environment in relation to their stress levels and well-being? 3. Are there particular spaces or features of the environment that have specific effects on stress levels and well-being across multiple participants? 4. How can the study results contribute to the nurses' well-being in practice and support evidence-based design in architecture? |
| **Methods** | | |  |
| Study design | 4 | Present key elements of study design early in the paper | Done. Please see the **Method and Analysis** section, the **Study Design** subsection where we explained the mixed-method approach - convergent parallel design. In addition, figure 1 displays a diagrammatic overview of the mixed-method approach in data collection with quantitative and qualitative methods applied. |
| Setting | 5 | Describe the setting, locations, and relevant dates, including periods of recruitment, exposure, follow-up, and data collection | Done. In the **Methods and Analysis** section, under **Study Setting** subsection, we outlined the details of the setting and location, as well as the relevant time period for program participation, recruitment procedures, and eligibility criteria. |
| Participants | 6 | Give the eligibility criteria, and the sources and methods of selection of participants | Done. In the **Methods and Analysis** section, under the **Study Participants** subsection, we outlined the details of the recruitment procedure and selection criteria. |
|  |  | For matched studies, give matching criteria and the number of controls per case | NA |
| Variables | 7 | Clearly define all outcomes, exposures, predictors, potential confounders, and effect modifiers. Give diagnostic criteria, if applicable | Done. Please see the **Method and Analysis** section, the following **Study Design** subsection. |
| Data sources/ measurement | 8* | For each variable of interest, give sources of data and details of methods of assessment (measurement). Describe comparability of assessment methods if there is more than one group | Done. Please see the **Method and Analysis** section, the following subsections: **Quantitative Data And Qualitative data** and **Data Analysis**. We provided sources for surveys and interview questions as supplementary material. |
| Bias | 9 | Describe any efforts to address potential sources of bias | Done. Please see the **Method and Analysis** section, **Ethical Considerations and Bias Control** subsection. Bias is addressed through anonymization, consistent data collection using predefined categories, participant transparency and control over their data, independent analysis by multiple researchers, and review of all data by hospital researchers to ensure accuracy and integrity. |
| Study size | 10 | Explain how the study size was arrived at | Done. In the **Methods and Analysis** section, under the **Study Participants** subsection, we outlined the details on how study size was arrived at.. The target sample size for this study is **20 participants**, determined by the use of comprehensive data collection methods, including interviews that provide rich objective and subjective insights. By employing purposeful sampling, the study ensures that selected individuals can offer valuable and detailed information, aligning with literature that supports smaller sample sizes in qualitative research (Sandelowski, 1996; Malterud et al., 2021; Zelčāne & Pipere, 2023) as sufficient for drawing significant conclusions. This is considered adequate because the depth and quality of the data collected can reveal nuanced insights that larger samples might not capture. |

| Quantitative variables | 11 | Explain how quantitative variables were handled in the analyses. If applicable, describe which groupings were chosen and why | Done. Please see the **Method and Analysis** section, **Quantitative data** and **Data Analysis** subsections. |
| --- | --- | --- | --- |
| Statistical methods | 12 | (*a*) Describe all statistical methods, including those used to control for confounding | Done. Please see the **Method and Analysis** section, **Data Analysis** subsection for detailed description of methods. Due to the large volume and variety of data to be collected, different statistical methods will be applied. These include cross-correlation analysis, Spearman's rank correlation, and multiple linear regression to appropriately analyse and interpret the diverse data types. |
|  |  | (*b*) Describe any methods used to examine subgroups and interactions | Done. Please see the **Method and Analysis** section, **Data Analysis** subsection for detailed description of methods. |
|  |  | (*c*) Explain how missing data were addressed | Done. Please see the **Method and Analysis** section, **Data Analysis** subsection for detailed description of methods. Missing data is addressed using methods like multiple imputation, LOCF, and sensitivity analyses for quantitative data. Qualitative data is analysed with available responses, noting limitations. Discrepancies are considered during triangulation to ensure accurate interpretation. Detailed explanation on how missing data is handled is available in the **Data Analysis** sub-section. |
|  |  | If applicable, explain how loss to follow-up was addressed | NA |
|  |  | (*e*) Describe any sensitivity analyses | NA |
| **Results** | | | |
| Participants | 13* | (a) Report numbers of individuals at each stage of study—eg numbers potentially eligible, examined for eligibility, confirmed eligible, included in the study, completing follow-up, and analysed | Done. Please see the **Method and Analysis** section, **Study Participants** subsection which explains that study aims for 20 participants who are medical staff, currently employed at the psychiatric healthcare facility and actively involved in their work activities. |
|  |  | (b) Give reasons for non-participation at each stage | NA |
|  |  | (c) Consider use of a flow diagram | NA |
| Descriptive data | 14* | (a) Give characteristics of study participants (eg demographic, clinical, social) and information on exposures and potential confounders | NA |
|  |  | (b) Indicate number of participants with missing data for each variable of interest | NA |
|  |  | (c) *Cohort study*—Summarise follow-up time (eg, average and total amount) | NA |
| Outcome data | 15* | Report numbers of outcome events or summary measures | NA |
| Main results | 16 | (*a*) Give unadjusted estimates and, if applicable, confounder-adjusted estimates and their precision (eg, 95% confidence interval). Make clear which confounders were adjusted for and why they were included | NA |
|  |  | (*b*) Report category boundaries when continuous variables were categorized | NA |
|  |  | (*c*) If relevant, consider translating estimates of relative risk into absolute risk for a meaningful time period | NA |

| Other analyses | 17 | Report other analyses done—eg analyses of subgroups and interactions, and sensitivity analyses | NA |
| --- | --- | --- | --- |
| **Discussion** | | | |
| Key results | 18 | Summarise key results with reference to study objectives | NA |
| Limitations | 19 | Discuss limitations of the study, taking into account sources of potential bias or imprecision. Discuss both direction and magnitude of any potential bias | Done. Please see the **Method and Analysis** section, **Discussion and Conclusion** subsection for limitations. As previously mentioned, bias was addressed in the Ethical **Considerations and Bias Control** sub-section. |
| Interpretation | 20 | Give a cautious overall interpretation of results considering objectives, limitations, multiplicity of analyses, results from similar studies, and other relevant evidence | NA |
| Generalisability | 21 | Discuss the generalisability (external validity) of the study results | Done. Please see the **Method and Analysis** section, **Discussion and Conclusion** subsection. |
| **Other information** | |  | |
| Funding | 22 | Give the source of funding and the role of the funders for the present study and, if applicable, for the original study on which the present article is based | The study did not receive any external funding. |

NA: Not applicable

COREQ (COnsolidated criteria for REporting Qualitative research) Checklist

| **Topic** | **Item No.** | **Guide Questions/Description** |
| --- | --- | --- |
| **Domain 1: Research team**  **and reflexivity** | | |
| *Personal characteristics* | | |
| Interviewer/facilitator | 1 | Which author/s conducted the interview or focus group?  **I #2 (MK)** - second author of the proposed mixed method study and one of the main investigators of the study  **C #1 (JF)** - university assistant, not author of the study, trained, and also with previous experience in conducting guided interviews  **C #2 (BM)** - university assistant, not author of the study, trained, and also with previous experience in conducting guided interviews |
| Credentials | 2 | What were the researcher’s credentials? E.g. PhD, MD  Authors:  **I #1 (MV)** - PhD  **I #2 (MK)** - PhD  **I #3 (FN)** - MD  **I #4 (MF)** - MD, PhD  Collaborators:  **C #1 (JF)** - MSc  **C #2 (BM)** - MSc  **C #3 (AD)** - MA |
| Occupation | 3 | What was their occupation at the time of the study?  Authors:  **I #1 (MV)** - Assistant Professor (Vienna University of Technology (TU Wien), Vienna, Austria)  **I #2 (MK)** - Assistant Professor (Vienna University of Technology (TU Wien), Vienna, Austria)  **I #3 (FN)** - MD, Dr., physician at the 2. Department for Psychiatry at Hietzing Clinic in Vienna, Austria  **I #4 (MF)** - MD, Prim. DDr., head of the 2. Department for Psychiatry at Hietzing Clinic in Vienna, Austria, head of the Karl Landsteiner Institute for Mental Health  Collaborators:  **C #1 (JF)** - Project Assistant (Vienna University of Technology (TU Wien), Vienna, Austria)  **C #2 (BM)** - PhD candidate (Vienna University of Technology (TU Wien), Vienna, Austria)  **C #3 (AD)** - MA (2. Department for Psychiatry at Hietzing Clinic in Vienna, Austria, Karl Landsteiner Institute for Mental Health) |
| Gender | 4 | Was the researcher male or female?  *Interviewers:* three female identifying  *Research team:* two females identifying, two male identifying |
| Experience and training | 5 | What experience or training did the researcher have?  **I #1 -** has experience in mixed methods research, experimental research and application of wearable sensor technology. She has an interest in healthcare design and healthcare architecture. She is EDAC certified.  **I #2 -** has experience in qualitative research and in conducting guided interviews in the healthcare setting (patients and staff). She has an interest in healthcare design and healthcare architecture. She is EDAC certified.  **I #3 -** is a psychiatrist with long experience in clinical practice in psychiatric hospitals. Furthermore, has experience in garden therapy and studying the effects of the environment on patient well-being.  **I #4 -** is a psychiatrist with long experience both in clinical practice and research. Has expertise in mixed-method research. Has experience with leading mixed-method research clinical studies.  **C #1 (JF)** - conducted observations and guided interviews for the master thesis research. She has received training for conducting observations and interviews for this study.  **C #2 (BM)** - conducts observations and guided interviews for the PhD thesis research. She has received training for conducting observations and interviews for this study.  **C #3 (AD)** - MA (2. Department for Psychiatry at Hietzing Clinic in Vienna, Austria, Karl Landsteiner Institute for Mental Health) |
| *Relationship with*  *participants* | | |
| Relationship established | 6 | Was a relationship established prior to study commencement?  **I #2 -** will conduct guided interviews.  **C #1 (JF)** - will conduct observations and guided interviews for the master thesis.  **C #2 (BM)** - will conduct guided interviews.  * None of the interviewers is involved in the recruitment process. Recruitment process is conducted by **I #1** and **C #3** |
| Participant knowledge of the interviewer | 7 | What did the participants know about the researcher? e.g. personal goals, reasons for doing the research  **I #2** will introduce herself to participants as an assistant professor at the Vienna University of Technology, and one of the investigators on the project. She will explain the guided interview procedure to participants and use an interview guide to facilitate the interview process.  **C #1** - will introduce herself to participants as a project assistant at the Vienna University of Technology and collaborator on the project, trained in conducting interviews. She will explain the guided interview procedure to participants and use an interview guide to facilitate the interview process.  **C #2** - will introduce herself to participants as a PhD candidate at the Vienna University of Technology and collaborator on the project, trained in conducting interviews. She will explain the guided interview procedure to participants and use an interview guide to facilitate the interview process.  Time of the introduction - Participants will be introduced to interviewers at the beginning of their study participation time.  Anticipated bias - see **Methods and Analysis** (Subsection **Qualitative Data**) and **Discussion and Conclusion** sections |
| Interviewer characteristics | 8 | What characteristics were reported about the interviewer/facilitator? e.g. Bias, assumptions, reasons and interests in the research topic  **I #2** is one the main investigators in the study, experience with qualitative research and guided interviews. **C #1** and **C #2** are under the supervision of **I #1** and **I #2** and they are trained in conducting guided interviews. |
| **Domain 2: Study design** | | |
| *Theoretical framework* | | |
| Methodological orientation and Theory | 9 | What methodological orientation was stated to underpin the study? e.g. grounded theory, discourse analysis, ethnography, phenomenology, content analysis  Please see the Introduction section for methodological orientation and theory. Please see the **Methods and Analysis** section for further methodological orientation. |
| *Participant selection* | | |
| Sampling | 10 | How were participants selected? e.g. purposive, convenience, consecutive, snowball  Please see **Methods and Analysis** section, **Study Participants** subsection. This section provides a detailed explanation of convenience sampling. |
| Method of approach | 11 | How were participants approached? e.g. face-to-face, telephone, mail, email  Please see **Methods and Analysis** section, **Study Participants** subsection. This section outlines the recruitment process. |
| Sample size | 12 | How many participants were in the study?  Please see **Methods and Analysis** section, **Study Participants** subsection. Study aims to recruit 20 participants. In the Methods and Analysis section, we justify the target sample size of 20 participants based on comprehensive data collection methods, including in-depth interviews. Purposeful sampling ensures participants provide valuable insights, supported by research on smaller sample sizes in qualitative studies (Sandelowski, 1996; Malterud et al., 2021; Zelčāne & Pipere, 2023), which are sufficient for meaningful conclusions. |
| Non-participation | 13 | How many people refused to participate or dropped out? Reasons?  NA |
| *Setting* | | |
| Setting of data collection | 14 | Where was the data collected? e.g. home, clinic, workplace  Please see **Methods and Analysis** section, subsection **Study Setting**. |
| Presence of non  participants | 15 | Was anyone else present besides the participants and researchers?  During interviews and surveys, no other individuals were present. However, during measurements involving wearable sensors and observations, participants were engaged in their usual activities, meaning other people were present in their environment. |
| Description of sample | 16 | What are the important characteristics of the sample? e.g. demographic data, date  Please see **Methods and Analysis** section, **Study Participants** subsection. |
| *Data collection* | | |
| Interview guide | 17 | Were questions, prompts, guides provided by the authors? Was it pilot tested?  Guided interview questions were provided by authors. See Supplementary file N. |
| Repeat interviews | 18 | Were repeat interviews carried out? If yes, how many?  No. |
| Audio/visual recording | 19 | Did the research use audio or visual recording to collect the data?  Please see **Methods and Analysis** section, subsection **Qualitative Data- Guided Interviews.** Interviews will be recorded and transcribed verbatim. |
| Field notes | 20 | Were field notes made during and/or after the interview or focus group?  Interviewers will take notes during and after each interview, documenting reminders, questions, thoughts, and interpretations. |
| Duration | 21 | What was the duration of the interviews or focus group?  Please see **Methods and Analysis** section, subsection **Qualitative Data- Guided Interviews**. Interviews are designed to last one hour. |
| Data saturation | 22 | Was data saturation discussed?  NA |
| Transcripts returned | 23 | Were transcripts returned to participants for comment and/or correction?  No |

| **Topic** | **Item No.** | **Guide Questions/Description** |
| --- | --- | --- |
| **Domain 3: analysis and**  **findings** | | |
| *Data analysis* | | |
| Number of data coders | 24 | How many data coders coded the data?  **C #1** and **C #2** will transcribe the interviews, with **I #2** reviewing recordings for accuracy and familiarizing with the data. **I #2** will then lead the coding process, identifying key patterns, while **I #1**, **I #3**, and **I #4** will review themes to ensure consistency and reliability. |
| Description of the coding tree | 25 | Did authors provide a description of the coding tree?  No. this will be reported in the findings publication. |
| Derivation of themes | 26 | Were themes identified in advance or derived from the data?  Please see Methods and Analysis section, Qualitative Data - Guided Interviews. Thematic analysis will be conducted afterwards, and the themes will be derived from data. More details will be available in the following publications, after the data is obtained and analyzed. |
| Software | 27 | What software, if applicable, was used to manage the data?  NA. More details will be available after data is collected and checked. |
| Participant checking | 28 | Did participants provide feedback on the findings?  NA |
| *Reporting* | | |
| Quotations presented | 29 | Were participant quotations presented to illustrate the themes/findings? Was each quotation identified? e.g. participant number  Please see the **Methods and Analysis** section, **Ethical Considerations and Bias Control** subsection. Yes, participant quotations will illustrate the themes and findings, with each identified by a participant number (e.g., "Participant 1" or "P1") to maintain anonymity. This approach ensures individual identities remain confidential while still providing valuable insights. Care will be taken to exclude any identifying information from the quotations. |
| Data and findings consistent | 30 | Was there consistency between the data presented and the findings?  NA. To be reported in the future publications, after data collection and analysis is finalized. |
| Clarity of major themes | 31 | Were major themes clearly presented in the findings?  NA. To be reported in the future publications, after data collection and analysis is finalized. |
| Clarity of minor themes | 32 | Is there a description of diverse cases or discussion of minor themes?  NA. To be reported in the future publications, after data collection and analysis is finalized. |

C: Collaborator; I: Investigator; NA: Not applicable; No.: Item reference number
