# Supplementary material for: Investigating the influence of the physical environment on psychiatric nurses wellbeing and professional interactions: A convergent parallel mixed-method study protocol
Source: PLoS One. 2026 Jan 15;21(1):e0340429. doi: 10.1371/journal.pone.0340429 (PMC12806848; doi:10.1371/journal.pone.0340429)
Supplement: S2 File — (DOCX) [file pone.0340429.s002.docx]

**S2 File. Semi-structured interview guide**

Interview questions are provided both in German and English

## **German:**

### **Interview Fragen:**

Allgemein:

1. Können Sie Ihr typisches Arbeitsumfeld auf der psychiatrischen Station beschreiben? *(Folge-/Überprüfungsfragen:Was sind die wichtigsten Merkmale oder Komponenten, die Ihnen auffallen? Haben Sie Änderungen an Ihrem Arbeitsumfeld vorgenommen? Wenn ja, warum?)*

2. Wie nehmen Sie den Einfluss der physischen Umgebung (z. B. Raumaufteilung, Beleuchtung, Möbel) auf Ihre tägliche Tätigkeit und die Interaktion mit den Patient*innen wahr?

Patient*innenbetreuung und Kommunikation:

3. Welche Aspekte des physischen Umfelds sind Ihrer Meinung nach am förderlichsten für eine qualitativ hochwertige Behandlung von psychiatrischen Patienten? (*Folge-/Überprüfungsfragen: Gibt es bestimmte Faktoren, die Sie bei Ihrer Arbeit als schwierig oder hinderlich empfinden? Wie würden Sie Ihre ideale Arbeitsumgebung für eine qualitativ hochwertige Pflege beschreiben?)*

4. Können Sie konkrete Erfahrungen oder Ereignisse nennen, bei denen die physische Umgebung Ihre Kommunikation/Interaktion mit Patient*innen beeinflusst hat? (positiv oder negativ)

5. Wie trägt Ihrer Meinung nach das physische Umfeld der psychiatrischen Klinik zum Wohlbefinden und zur Genesung der Patienten bei?
*(Folge-/Überprüfungsfragen: Gibt es Verbesserungsmöglichkeiten, die Ihrer Meinung nach die Behandlungsergebnisse und/oder das Wohlbefinden der Patient*innen verbessern könnten? Wenn Sie eine Sache in der physischen Umgebung ändern könnten, um die Patient*innenversorgung und/oder die Kommunikation mit den Patient*innen zu verbessern, was wäre das?)*

Zusammenarbeit, Kommunikation und Arbeitszufriedenheit des Teams:

6. Können Sie konkrete Erfahrungen oder Ereignisse schildern, bei denen die physische Umgebung Ihre Kommunikation mit anderem medizinischen Personal oder Ihre allgemeine Arbeitsleistung beeinflusst hat? (positiv oder negativ)

7. Wie wirkt sich Ihrer Meinung nach das physische Umfeld auf die Arbeitsmoral, das Stressniveau und die allgemeine Arbeitszufriedenheit des Personals auf der psychiatrischen Station aus?
*(Folge-/Überprüfungsfragen: Gibt es etwas, das Sie an der physischen Umgebung ändern würden, um den Stress zu verringern und/oder die Arbeitsmoral und Zufriedenheit der Mitarbeiter*innen zu verbessern?)*

8. Welches sind einige der wichtigsten Herausforderungen oder Einschränkungen, mit denen Sie in der gegenwärtigen physischen Umgebung der psychiatrischen Klinik konfrontiert sind, und wie wirken sich diese auf die Effektivität der Erfüllung Ihrer Aufgaben aus?
*(Folge-/Überprüfungsfragen: Wie könnte Ihrer Meinung nach die physische Umgebung verbessert werden, um die Bedürfnisse des Personals besser zu unterstützen?)*

Weitere:

9. Gab es in letzter Zeit Änderungen oder Renovierungen in der physischen Umgebung der psychiatrischen Station?
*(Folge-/Überprüfungsfrage: Wenn ja, wie wurden diese Änderungen vom Personal und den Patient*innen wahrgenommen? Wenn nein, sind Änderungen geplant und warum? Planen Sie und Ihre Kolleg*innen (als Alltagsgestalter*innen), Änderungen an Ihrer Umgebung vorzunehmen?)*

10. Gibt es bestimmte Ressourcen oder Unterstützungsmechanismen, die Ihrer Meinung nach hilfreich wären, um Herausforderungen im Zusammenhang mit der physischen Umgebung der psychiatrischen Station zu bewältigen?

11. Haben Sie Änderungen Ihres Befindens festgestellt, wenn Sie zwischen verschiedenen Bereichen des Gartens umher gehen?

12. Erzeugen verschiedene Umgebungen innerhalb des Gartens unterschiedliche körperliche Empfindungen? Beschreiben Sie bitte, was Sie wahrgenommen haben?

13. Haben Sie sich in bestimmten Bereichen eher entspannt oder eher gestresst gefühlt? Was hat Ihrer Meinung nach zu diesem Gefühl beigetragen?

**English:**

### **Interview Questions:**

#### General:

1. Can you describe your typical work environment on the psychiatric ward?
   *(Follow-up/probing questions: What are the key features or components that stand out to you? Have you made any changes to your work environment? If so, why?)*
2. How do you perceive the impact of the physical environment (e.g., room layout, lighting, furniture) on your daily tasks and interactions with patients?

#### Patient Care and Communication:

1. In your opinion, which aspects of the physical environment are most conducive to providing high-quality care for psychiatric patients?
   *(Follow-up/probing questions: Are there any specific factors you find challenging or obstructive in your work? How would you describe your ideal work environment for high-quality care?)*
2. Can you share specific experiences or events where the physical environment influenced your communication or interaction with patients? *(positively or negatively)*
3. In your opinion, how does the physical environment of the psychiatric clinic contribute to patient well-being and recovery?
   *(Follow-up/probing questions: Are there any improvements you believe could enhance treatment outcomes and/or patient well-being? If you could change one aspect of the physical environment to improve patient care and/or communication with patients, what would it be?)*

#### Team Collaboration, Communication, and Job Satisfaction:

1. Can you describe any specific experiences or situations where the physical environment affected your communication with other medical staff or your overall job performance? *(positively or negatively)*
2. How do you think the physical environment influences staff morale, stress levels, and overall job satisfaction on the psychiatric ward?
   *(Follow-up/probing questions: Is there anything you would change in the physical environment to reduce stress and/or improve staff morale and job satisfaction?)*
3. What are some of the main challenges or limitations you face in the current physical environment of the psychiatric clinic, and how do these affect your ability to perform your duties effectively?
   *(Follow-up/probing questions: How do you think the physical environment could be improved to better support staff needs?)*

#### Additional Questions:

1. Have there been any recent changes or renovations in the physical environment of the psychiatric ward?
   *(Follow-up/probing questions: If so, how were these changes perceived by staff and patients? If not, are any changes planned, and why? Do you and your colleagues (as everyday users of the space) plan to make any modifications to your environment?)*
2. Are there any specific resources or support mechanisms that you believe would be helpful in addressing challenges related to the physical environment of the psychiatric ward?
3. Have you noticed any changes in your well-being when moving through different areas of the garden?
4. Do different environments within the garden evoke different physical sensations? Please describe what you have experienced.
5. Did you feel more relaxed or more stressed in certain areas of the garden? What do you think contributed to this feeling?
