# Supplementary material for: Investigating the influence of the physical environment on psychiatric nurses wellbeing and professional interactions: A convergent parallel mixed-method study protocol
Source: PLoS One. 2026 Jan 15;21(1):e0340429. doi: 10.1371/journal.pone.0340429 (PMC12806848; doi:10.1371/journal.pone.0340429)
Supplement: Attachment — Submitted filename: Revision of PONE-D-25-06162.docx [file pone.0340429.s003.docx]

**Revision of PONE-D-25-06162**

The manuscript presents an original study protocol investigating how the physical environment affects psychiatric nurses’ well-being and professional interactions. The manuscript is clearly structured, written in intelligible English, and adheres to accepted ethical and reporting standards.

Suggested improvements include refining sentence structure in the methods section, ensuring consistent terminology, and correcting occasional grammatical issues. Once these major revisions are made, the article will be suitable for publication.

**Originality and Relevance**

The study protocol introduces a novel interdisciplinary approach, focusing specifically on psychiatric nurses. While environmental factors have been examined in other healthcare contexts, the direct link between spatial features and psychiatric nurses’ stress levels and interactions remains insufficiently studied. The manuscript therefore contributes meaningfully to current knowledge and future practice.

**Methods and Analyses**

The methodology is rigorous and well described. The use of wearable sensors, systematic observations, surveys, and interviews ensures a robust mixed-methods design. Ethical considerations, data security, and participant confidentiality are carefully addressed. The description of instruments and procedures is detailed enough for replication. Statistical analyses and qualitative approaches are appropriate.

However, I think the authors should add a semi-structured qualitative interview protocol suggesting which questions would be asked, how interviewers’ observation will be integrated, and the characteristics of the devices in which interviews will be recorded. Interviewers’ characteristics should also be discussed to address potential biases.

Additionally, while the quantitative and, to a lesser extent, qualitative parts of this study are described, I suggest the reviewers add a section describing how will integration of both types of outcomes will be conducted, as this is an integral part of mixed-methods approaches which is currently missing. I would recommend the authors read this article for further guidance: Playful experience, learning perception, and motivation in a cooperative escape room activity in undergraduate nursing education: A mixed-methods study. https://doi.org/10.1016/j.learninstruc.2025.102212.

Minor language polishing in the methods section would improve readability, particularly when describing sensor use and environmental measurements.

**Results and Conclusions**

As this is a study protocol, no results are yet reported. The expected outcomes are clearly articulated and align with the stated research questions. The conclusions appropriately emphasize the study’s contribution to future evidence-based design in psychiatric hospitals. They are cautious, proportionate, and supported by the proposed methodology.

**Presentation and Clarity**

The manuscript is generally well written in standard English. Certain passages are dense, especially in the methods section, and could benefit from shorter sentences to improve flow. Figures and tables are useful and informative. References are comprehensive and up to date. Minor grammatical adjustments and stylistic refinements will further strengthen clarity.

**Ethics and Integrity**

The study has received ethical approval from the appropriate ethics committee. Procedures for informed consent, anonymization, and bias control are rigorous and in line with international standards. The manuscript also commits to open data principles, ensuring research transparency.

**Data Availability and Reporting Standards**

The protocol clearly states how data will be made available upon completion of the study. The authors show adherence to relevant reporting guidelines (GRAMMS, STROBE, COREQ). Community standards for reproducibility and transparency are respected.
