# Supplementary material for: Investigating the influence of the physical environment on psychiatric nurses wellbeing and professional interactions: A convergent parallel mixed-method study protocol
Source: PLoS One. 2026 Jan 15;21(1):e0340429. doi: 10.1371/journal.pone.0340429 (PMC12806848; doi:10.1371/journal.pone.0340429)
Supplement: Attachment — Submitted filename: Response to Reviewers.docx [file pone.0340429.s006.docx]

PONE-D-25-06162

Investigating the Influence of the Physical Environment on Psychiatric Nurses Well-Being and Professional Interactions: A Convergent Parallel Mixed-Method Study Protocol

PLOS ONE

Response to Reviewers

Dear Editors and Reviewers,

Thank you for your comments. The following document details all the changes made in the manuscript "Investigating the Influence of the Physical Environment on Psychiatric Nurses Well-Being and Professional Interactions: A Convergent Parallel Mixed-Method Study Protocol" according to editors’ and reviewers’́ comments.

**Major revisions: List of changes**

| Editor | Response and changes |
| --- | --- |
| Thank you for submitting your manuscript to PLOS ONE. After careful consideration, we feel that it has merit but does not fully meet PLOS ONE’s publication criteria as it currently stands. Therefore, we invite you to submit a revised version of the manuscript that addresses the points raised during the review process. | Thank you for the opportunity to revise our manuscript. We have carefully read and considered all comments and suggestions provided by the editors and reviewers. Each point has been addressed in detail, and the manuscript has been thoroughly revised to reflect these improvements. We hope the revised version meets the journal’s expectations and publication criteria. |
| 1. Please ensure that your manuscript meets PLOS ONE's style requirements, including those for file naming. The PLOS ONE style templates can be found at  <https://journals.plos.org/plosone/s/file?id=wjVg/PLOSOne_formatting_sample_main_body.pdf> and  [https://journals.plos.org/plosone/s/file?id=ba62/PLOSOne_formatting_sample_title_authors_affiliations.pdf](https://track.editorialmanager.com/CL0/https:%2F%2Fjournals.plos.org%2Fplosone%2Fs%2Ffile%3Fid=ba62%2FPLOSOne_formatting_sample_title_authors_affiliations.pdf/1/010f0199e571930e-8c098444-ba11-4753-927e-1ce0ff32d952-000000/dNbzpW6ToEuPRvKSXtcyEngQ2M2E65G7W5QjLel5prg=233) | Thank you for providing links for PLOS ONE style templates. We have carefully revised templates and guidelines and revised our manuscript accordingly. |
| 2. We note that the grant information you provided in the ‘Funding Information’ and ‘Financial Disclosure’ sections do not match.  When you resubmit, please ensure that you provide the correct grant numbers for the awards you received for your study in the ‘Funding Information’ section. | Thank you for noting this discrepancy. We have provided clarification regarding the funding information and corrected details in the cover letter, as suggested, to ensure consistency. |
| 3. Thank you for stating the following financial disclosure:  “TU Wien Bibliothek provides financial support through its Open Access Funding Programme.”  Please state what role the funders took in the study. If the funders had no role, please state: "The funders had no role in study design, data collection and analysis, decision to publish, or preparation of the manuscript."  If this statement is not correct you must amend it as needed.  Please include this amended Role of Funder statement in your cover letter; we will change the online submission form on your behalf. | Thank you for bringing to our attention needed revisions on financial disclosure. We have added to “Cover Letter” the statement:  *"TU Wien Bibliothek provides financial support through its Open Access Funding Programme. The funders (TU Wien Bibliothek) had no role in study design, data collection and analysis, decision to publish, or preparation of the manuscript."* |
| 4. In the online submission form, you indicated that “No datasets were generated or analysed during the current study. All relevant data from this study will be made available upon study completion.”  All PLOS journals now require all data underlying the findings described in their manuscript to be freely available to other researchers, either 1. In a public repository, 2. Within the manuscript itself, or 3. Uploaded as supplementary information.  This policy applies to all data except where public deposition would breach compliance with the protocol approved by your research ethics board. If your data cannot be made publicly available for ethical or legal reasons (e.g., public availability would compromise patient privacy), please explain your reasons on resubmission and your exemption request will be escalated for approval. | Thank you for clarifying the data availability requirements. This submission presents the research study protocol and not the results of an already conducted research study. Therefore, the dataset is not yet available because data processing is still ongoing. Once data are retrieved from the sensors and processed (in .csv and .txt formats), all data underlying the findings described in this study will be made freely available in a public repository to other researchers in accordance with PLOS data sharing policies. |
| 5. When completing the data availability statement of the submission form, you indicated that you will make your data available on acceptance. We strongly recommend all authors decide on a data sharing plan before acceptance, as the process can be lengthy and hold up publication timelines. Please note that, though access restrictions are acceptable now, your entire data will need to be made freely accessible if your manuscript is accepted for publication. This policy applies to all data except where public deposition would breach compliance with the protocol approved by your research ethics board. If you are unable to adhere to our open data policy, please kindly revise your statement to explain your reasoning and we will seek the editor's input on an exemption. Please be assured that, once you have provided your new statement, the assessment of your exemption will not hold up the peer review process. | Thank you for your guidance regarding the data sharing plan. This manuscript outlines a research protocol, and it does not contain any research results. When the study is completed, the data will be made available following processing and preparation (retrieved from sensors and organised in .csv and .txt formats). Once finalised, the complete dataset underlying the findings will be deposited in a public repository (e.g., the university’s institutional open data repository, Zenodo etc.) and made freely accessible to other researchers, in full accordance with PLOS data availability policies.  We have also reflected this in the cover letter, stating:  “*All underlying data from this study will be made freely accessible in a public repository once processed and prepared, in compliance with PLOS data sharing requirements.*” |
| 6. Please ensure that you include a title page within your main document. You should list all authors and all affiliations as per our author instructions and clearly indicate the corresponding author. | Title page is now included within the main document. |
| 7. If the reviewer comments include a recommendation to cite specific previously published works, please review and evaluate these publications to determine whether they are relevant and should be cited. There is no requirement to cite these works unless the editor has indicated otherwise. | In one instance, the reviewer asked us to review a specific paper to understand better how to explain our methodology. We carefully examined the suggested publication as an example to improve the description of the convergent parallel design used in our study. Since the reviewer did not request a citation or inclusion of material from that paper, we did not cite it. However, we have revised the *Methods and Analysis* section to include additional clarifications as recommended by the reviewer. |

| Reviewer #1 | Response and changes |
| --- | --- |
| The manuscript presents an original study protocol investigating how the physical environment affects psychiatric nurses’ well-being and professional interactions. The manuscript is clearly structured, written in intelligible English, and adheres to accepted ethical and reporting standards.  Suggested improvements include refining sentence structure in the methods section, ensuring consistent terminology, and correcting occasional grammatical issues. Once these major revisions are made, the article will be suitable for publication. | Thank you for your detailed and insightful review. The revised manuscript has carefully addressed issues that were brought to our attention. We have, through revised text, addressed refining sentence structure in the methods section, ensuring consistent terminology, and correcting occasional grammatical issues. Please find the answers in the following paragraphs. |
| **Originality and Relevance**  The study protocol introduces a novel interdisciplinary approach, focusing specifically on psychiatric nurses. While environmental factors have been examined in other healthcare contexts, the direct link between spatial features and psychiatric nurses’ stress levels and interactions remains insufficiently studied. The manuscript therefore contributes meaningfully to current knowledge and future practice. | We thank the reviewer for this positive and encouraging feedback, and we appreciate the recognition of the study’s contribution to advancing knowledge on environmental influences in psychiatric nursing practice. |
| The methodology is rigorous and well described. The use of wearable sensors, systematic observations, surveys, and interviews ensures a robust mixed-methods design. Ethical considerations, data security, and participant confidentiality are carefully addressed. The description of instruments and procedures is detailed enough for replication. Statistical analyses and qualitative approaches are appropriate.  However, I think the authors should add a semi-structured qualitative interview protocol suggesting which questions would be asked, how interviewers’ observation will be integrated, and the characteristics of the devices in which interviews will be recorded. Interviewers’ characteristics should also be discussed to address potential biases. | Thank you very much for this helpful feedback on how to better describe the guided interview component of our study. To address your comments, we have added the major topics in the interview guide in the manuscript text, as well as the complete interview guide in the supplementary material. In this study, we do not systematically record interviewers’ observations; the analysis is done on verbatim transcripts only. We have made this explicit in the Methods (section Qualitative Data - Guided Interviews) and noted it as a limitation of the study (in Discussion and Conclusion). We also added additional information about the recording devices and interviewers’ characteristics in the section Qualitative Data - Guided Interviews. |
| Additionally, while the quantitative and, to a lesser extent, qualitative parts of this study are described, I suggest the reviewers add a section describing how will integration of both types of outcomes will be conducted, as this is an integral part of mixed-methods approaches which is currently missing. I would recommend the authors read this article for further guidance: Playful experience, learning perception, and motivation in a cooperative escape room activity in undergraduate nursing education: A mixed-methods study. https://doi.org/10.1016/j.learninstruc.2025.102212. | Thank you for the reference paper. It helps us understand how to better explain convergent parallel design used in our study and how the analysis process will be developed based on created datasets.  We added in lines 189-196: *“Each dataset is analysed separately using methods suited to its format, i.e., statistical analysis for sensor and physiological data, and thematic coding for interview and observational material. Findings are then aligned through joint displays and comparison matrices to identify convergence, complementarity, or discrepancy, supporting an integrated interpretation grounded in both experiential and objective perspectives. This analytical structure enables environmental measures and physiological indicators to be directly compared with staff experiences and perceptions, ensuring that subjective reports are neither assumed nor dismissed, but rather examined alongside empirical observations.”* |
| Minor language polishing in the methods section would improve readability, particularly when describing sensor use and environmental measurements. | We thank the reviewer for this helpful suggestion. The language in the Methods section, particularly in the parts describing sensor use and environmental measurements, has been carefully revised to improve clarity and readability. Sentences are added throughout sub-sections **Wearable Sensor Deployment** and **Physical Environment Measurement and Documentation** |
| **Results and Conclusions**  As this is a study protocol, no results are yet reported. The expected outcomes are clearly articulated and align with the stated research questions. The conclusions appropriately emphasize the study’s contribution to future evidence-based design in psychiatric hospitals. They are cautious, proportionate, and supported by the proposed methodology. | We thank the reviewer for the positive and encouraging feedback. |
| **Presentation and Clarity**  The manuscript is generally well written in standard English. Certain passages are dense, especially in the methods section, and could benefit from shorter sentences to improve flow. Figures and tables are useful and informative. References are comprehensive and up to date. Minor grammatical adjustments and stylistic refinements will further strengthen clarity. | We thank the reviewer for their positive and helpful feedback. The **Methods** section and other dense passages have been carefully revised to improve readability, with several sentences shortened for better flow. Grammatical and stylistic adjustments have also been made throughout the manuscript to enhance clarity and overall presentation. |
| **Ethics and Integrity**  The study has received ethical approval from the appropriate ethics committee. Procedures for informed consent, anonymization, and bias control are rigorous and in line with international standards. The manuscript also commits to open data principles, ensuring research transparency. | We appreciate the acknowledgement of the study’s adherence to ethical standards and commitment to transparency through the open data principles. |
| **Data Availability and Reporting Standards**  The protocol clearly states how data will be made available upon completion of the study. The authors show adherence to relevant reporting guidelines (GRAMMS, STROBE, COREQ). Community standards for reproducibility and transparency are respected. | We appreciate the reviewer’s recognition of our compliance with reporting standards and commitment to reproducibility and transparency. |

| Reviewer #2 | Response and changes |
| --- | --- |
| The study protocol titled “Investigating the Influence of the Physical Environment on Psychiatric Nurses Well-Being and Professional Interactions: A Convergent Parallel Mixed-Method Study Protocol” is well-written and informative. Below are some comments (mostly statistical) to the authors: | We appreciate the reviewer’s careful assessment and helpful suggestions. The revised manuscript has carefully addressed issues that were brought to our attention. Please find the answers in the following paragraphs. |
| 1. Line 154: Can you please elaborate on what sense the methodology is reproducible? If the methodology is using convenience sampling, can this be reproducible? Even if it was using any statistical sampling technique, the same study participants may not be repeatedly selected for reproducibility purposes. | We thank the reviewer for this valuable comment and for the opportunity to clarify the meaning of reproducibility in our study. We fully agree that, given the use of convenience and purposeful sampling, the same participants cannot be repeatedly selected, and statistical replication is not implied. In the revised manuscript, we have clarified that reproducibility refers specifically to the methodological reproducibility of the study. Namely, the ability of other researchers to replicate the procedures, instruments, and analytical framework under comparable conditions. Detailed documentation of data collection tools and analytic procedures ensures transparency and allows the study design to be replicated or adapted in other psychiatric or healthcare contexts, even when participant samples differ. We believe this clarification now more accurately reflects the scope of reproducibility intended in our research.  Revisions have been made throughout the Methods and Analysis section. Some of the newly added paragraphs are as follows:  Section **Method and Analysis**  Subsection **Study Design**  *In this context, reproducibility refers to the capacity of other researchers to replicate the procedural design, measurement instruments, and analytical approach, rather than to reproduce the same participant sample or identical datasets. While convenience and purposeful sampling inherently constrain statistical replication, the study ensures procedural transparency through detailed documentation of all protocols, sensor configurations, and analytic workflows. This methodological clarity enables the study methodology to be reapplied, tested, or adapted across comparable psychiatric or healthcare settings, thereby supporting consistency and comparability of results despite variations in sample composition. Therefore, this approach supports the scalability of the study protocol and its adaptation to different contexts and settings.*  Section **Method and Analysis**  Subsection **Study Design**  *To ensure methodological transparency and reproducibility, the study follows clearly defined, stepwise procedures for data collection, processing, and analysis. All instruments, sensor configurations, and survey tools are standardized and documented to allow replication in other psychiatric or healthcare environments. The mixed-methods framework, with its parallel yet integrable data streams, supports consistent replication across settings with different spatial or organizational characteristics. Moreover, data handling protocols, ethical safeguards, and analytic procedures are designed to be adaptable, enabling other research teams to reproduce or extend the study while maintaining comparability of results. This systematic structure enhances scientific credibility and facilitates cumulative knowledge-building in environmental and mental health research.*  *…add more from other parts/sub-sections* |
| 2. Line 169: Four different units out of how many total units? What are the reasons for choosing only four units and why/how they were chosen? Please clarify. | Thank you for bringing this to our attention. The four listed units are at the same time all units of the psychiatric clinic where the study was conducted. This is now corrected in the revised manuscript:  Section **Method and Analysis**  Subsection **Study Setting**  *“The study is conducted in the 2. Department of Psychiatry at Hietzing Clinic in Vienna, Austria, which consists of four different units and involves nursing staff, in the period between May 2024 and May 2025.”* |
| 3. Line 170: any specific reason for writing these age groups separately, instead of "age 18 and above"? | Thank you for pointing out that this explanation was unclear. The age groups were specified because three out of four units admit patients belonging to distinct age categories. This information is obtained from the psychiatric clinic where the study took place. The text has been revised accordingly to clarify this distinction:  Section **Method and Analysis**  Subsection **Study Setting**  *“All four units are involved in the study: 1) a unit for acute psychiatric admissions for patients aged 18-49, 2) a unit for acute psychiatric admissions for patients aged 50 and above, 3) a transpsychiatric unit for patients aged 16-25 and 4) a unit for people with intellectual disability and psychiatric disorders.”* |
| 4. Line 172: By "...all units", did you mean the four selected units? Please, paraphrase for clarity if so. | The four listed units represent all the units of the psychiatric clinic where the study was conducted. This has been corrected in the revised manuscript:  Section **Method and Analysis**  Subsection **Study Setting**  *“The study is conducted in the 2. Department of Psychiatry at Hietzing Clinic in Vienna, Austria, which consists of four different units and involves nursing staff, in the period between May 2024 and May 2025.”*  Corrections have also been made throughout the manuscript wherever the department units are mentioned, to improve clarity. |
| 5. Line 200: In the supplement, the authors mentioned “In the Methods and Analysis section, we justify the target sample size of 20 participants based on comprehensive data collection methods, including in-depth interviews.” However, this justification falls short of a rigorous statistical approach. Please elaborate on how the sample size is justifiable in the absence of any statistical hypothesis, related power analysis, and potential participant dropout considerations. | We thank the reviewer for this insightful comment and for highlighting the need for a clearer justification of the sample size. We agree that, given the absence of a formal statistical hypothesis, a traditional power analysis was not applicable. The revised manuscript now provides an expanded rationale for the target of 20 participants. Specifically, we emphasize that the sample size was determined based on methodological considerations rather than statistical inference, reflecting the study’s exploratory, mixed-methods nature. Each participant contributes extensive physiological, environmental, and qualitative data, allowing for analytical depth and triangulation. The justification now references established qualitative concepts of information power and data saturation (Sandelowski, 1996; Malterud et al., 2016), which support the adequacy of smaller, information-rich samples. We have also clarified that the target size accounts for potential participant dropout within the study timeline.  The revised text in the manuscript and supplementary files respectively, is as follows:  Manuscript:  Supplementary file:  *“The study aims to recruit 20 participants, a sample size determined by methodological rather than statistical considerations. Given the exploratory, mixed-methods design and the intensive data collection process, including continuous physiological monitoring, environmental sensing, and in-depth qualitative interviews, each participant contributes a substantial volume of both quantitative and qualitative data. This approach prioritises data richness and analytical depth over statistical generalizability.*  *Although no formal power analysis was conducted due to the absence of a predefined statistical hypothesis, the target number aligns with qualitative research principles of information power and data saturation, which suggest that smaller, well-defined samples can yield meaningful and transferable findings when each case is thoroughly examined (Sandelowski, 1996; Malterud et al., 2016; Zelčāne & Pipere, 2023). Furthermore, the sample size accounts for the potential of minor participant attrition, as recruitment is ongoing and replacement of withdrawn participants is feasible within the study period. This strategy ensures sufficient data for robust within- and cross-case analyses while maintaining feasibility in a sensitive psychiatric hospital setting.”* |

| Reviewer #3 | Response and changes |
| --- | --- |
| This is a very interesting write up. The abstract was very enlightening you did a good job on it. | We appreciate the reviewer’s time and effort in evaluating our manuscript. |
| 1. In the Study Setting, can you be more detailed in the explanation of the study setting | Additional details have been incorporated into the Study Setting subsection, providing a more comprehensive description of the units within the psychiatric clinic where the study is conducted. We have also elaborated on the researchers’ position in relation to staff–patient interactions, clinical routines, and the procedures for data collection. |
| 2. In Study Participants line 218 and 219 don't seem together or continuous kindly check. | The Study Participants subsection was substantially revised to provide clearer explanations and enhance the overall readability and continuity of the section. Specific lines, indicated by the reviewer were addressed with this revision. |
| 3. Can you explain what you mean in Line 243 and possibly rephrase? | We appreciate the reviewer’s observation regarding the lack of clarity in line 243. The line and its surrounding text have been revised to more clearly describe how shifts are structured and how observations are organized in relation to them. The revised text is presented below:  “*Each nursing shift lasts between 8 and 12 hours, depending on the nurse’s role within the ward hierarchy and their specific clinical responsibilities. To obtain a minimum of 20 hours of valid heart rate recordings and observational data during active shifts, and to capture reliable measures of sleep and light exposure over a continuous two-week period, an individualized data-collection schedule is developed for each participant. An example of the schedule for one participant is shown in Fig 2. This tailored scheduling approach ensures balanced coverage of different shift types (day, evening, and night). It accounts for variations in workload and rest periods, thereby improving data consistency and minimising missing or biased measurements.*” |
| 4. Kindly replace insure with ensure | Thank you for your detailed review and for pointing out this mistake. We have revised the wording accordingly. |
| 5. With a writeup of this quality there needs to me more tidying with the result and if possible, recommendations which was not included. The article would be more qualitative with those included. Thank you | Thank you for your valuable comment. As this paper presents the study protocol, specific results are not yet available; however, we have expanded the text to clarify the anticipated outcomes. These additions also emphasize how the results will inform practical improvements in psychiatric facility design and future research directions. |
