# Supplementary material for: Investigating the influence of the physical environment on psychiatric nurses wellbeing and professional interactions: A convergent parallel mixed-method study protocol
Source: PLoS One. 2026 Jan 15;21(1):e0340429. doi: 10.1371/journal.pone.0340429 (PMC12806848; doi:10.1371/journal.pone.0340429)
Supplement: Attachment — Submitted filename: Revision of PONE-D-25-06162-R1.docx [file pone.0340429.s005.docx]

**Revision of PONE-D-25-06162-R1**

The revised manuscript presents a well-structured and methodologically robust study protocol. The authors have made substantial improvements in clarity, methodological transparency, and integration of mixed-methods procedures. The manuscript now adheres closely to reporting standards and demonstrates clear potential to contribute meaningfully to interdisciplinary research in mental-healthcare design.

**Originality and Relevance**

The study addresses an understudied intersection between architectural environments and psychiatric nursing practice. The interdisciplinary framework is well justified, and the enhancements introduced in this revision strengthen the conceptual and practical relevance of the work.

**Methods and Analyses**

The authors have significantly improved the methodological clarity of the protocol. Notably:

The qualitative section now includes detailed explanations of interviewer characteristics, recording devices, and steps taken to minimize bias.

The mixed-methods integration approach is now clearly articulated with explicit mention of joint displays and comparison matrices.

The Study Setting and Study Participants sections have been reorganized to enhance continuity and precision.

The justification of sample size has been expanded following established qualitative principles (information power, data saturation).

Quantitative and qualitative analytic strategies are well defined, and triangulation procedures are transparent and coherent.

**Results and Expected Contributions**

As this is a protocol paper, results are not yet expected. The anticipated outcomes are now more clearly linked to the research questions and methodological design. The study's potential to inform evidence-based psychiatric facility design is well articulated.

**Presentation and Style**

Revisions have improved readability throughout, particularly in the Methods section. Sentences have been shortened or reorganized, terminology is now consistent, and descriptions of measurement instruments and procedures are clearer. Figures, tables, and supplementary materials effectively support transparency and reproducibility.

**Ethics and Reporting Standards**

Ethical considerations, anonymization procedures, and responsible research safeguards are thoroughly described. Adherence to GRAMMS, STROBE, and COREQ is evident. Data availability procedures comply with PLOS ONE guidelines.

**Final Recommendation**

The manuscript is now suitable for publication, as the authors have comprehensively addressed all prior reviewer concerns. The current version represents a significant improvement in clarity, methodological transparency, and scholarly rigor.
